# Consumer Preferences and Assortment in Large-Scale Retail of Lamb Meat: A Comparative Study in the Metropolitan Area of Turin (North-West Italy)

**DOI:** 10.3390/foods15040703

**Published:** 2026-02-13

**Authors:** Chiara Costamagna, Valentina Maria Merlino, Danielle Borra, Stefano Massaglia, Gullì Carmine Giuseppe, Antonio Mimosi, Paolo Cornale

**Affiliations:** Department of Agricultural, Forest and Food Sciences, University of Turin, Largo Baccini 2, 10095 Grugliasco, TO, Italy; c.costamagna@unito.it (C.C.); danielle.borra@unito.it (D.B.); stefano.massaglia@unito.it (S.M.); carmine.gulli@unito.it (G.C.G.); antonio.mimosi@unito.it (A.M.); paolo.cornale@unito.it (P.C.)

**Keywords:** choice analysis, lamb supply, consumer decision-making, seasonal sale

## Abstract

This study introduced two distinct investigations conducted in a specific area of Italy (North-West) in order to analyses and compare the supply and demand of lamb meat. The first involved a survey using a questionnaire administered to 135 consumers in the metropolitan area of Turin, examining their choices and preferences, as well as the reasons why 212 non-consumers avoid lamb meat. Concurrently, a study was carried out in the sales points of large-scale retail (LSR) in Turin, focusing on the attributes used to market lamb meat. By comparing the results of the consumer survey, conducted using the Best-Worst Scaling method, with the analysis of LSR offerings, it was found that consumer preferences are only partially aligned with the product offerings. The mismatch between LSR supply and demand is significant in highlighting potential inefficiencies along the supply chain and opportunities not fully exploited by the distribution system. For example, the increase in product availability during the festive period contrasts with consumers’ non-seasonal consumption. Even the lack of organic certification in LSR products contrasts with consumer preferences. However, the wide variety of product origins and the availability of different meat cuts align well with consumer preferences. These findings can inform marketing strategies in large retail chains, enabling them to better align with consumer choices.

## 1. Introduction

In recent years, the market and production trends for sheep meat have shown a consistent decline across Europe and Italy [1,2]. This decrease is characterized by a reduction in the number of animals and farming enterprises, leading to increased imports of live animals from countries such as Hungary, Romania, and Spain [3]. Concurrently, consumption and purchasing of sheep meat have also decreased, relegating lamb meat to the status of a niche product compared to more commonly consumed meats [4]. In fact, as reported in [4], the global small ruminant sector comprised approximately 1209 million sheep and 1046 million goats, accounting for slightly less than 12% of total livestock units worldwide. Lamb is often associated with religious celebrations and traditional events [5], rendering it a seasonal food in specific geographic contexts. In Italy, lamb consumption is particularly linked to Christian festivities such as Easter and Christmas [6], with this pattern being more pronounced in certain northern regions compared to the south, where lamb traditions extend beyond festive occasions [7].

The declining trend in lamb meat consumption can be attributed to several factors [8]. First, growing consumer awareness of animal welfare and ethical concerns regarding the consumption of animal-derived products have influenced purchasing behaviors, often favoring plant-based alternatives [9,10] over cultural and culinary traditions. In the specific case of lamb, the slaughter of young animals is particularly impactful on public sentiment [11], a concern exacerbated by animal rights campaigns.

Other contributing factors include the high price of lamb meat, challenges in sourcing and preparing it, and the limited availability of processed and ready-to-eat lamb products [5]. According to [12], purchasing behavior is influenced by socio-demographic factors: younger individuals and those with lower incomes are less willing to pay a premium price, while women prioritize ease of preparation, especially cooking time [13]. Developing processed lamb-based products could address the preparation challenges and potentially stimulate consumption. However, these products remain scarce and less well-known compared to processed products from veal, pork, and chicken, with the exception of baby food [14].

Sheep production—including meat, dairy, and wool—represents a vital part of the environmental, cultural, and culinary heritage of Mediterranean regions [15]. The European Union has recognized this importance by granting Protected Geographical Indication (PGI) status to three types of Italian lamb meat: *Agnello Sardo*, *Abbacchio Romano*, and *Agnello del Centro Italia* [16]. PGI certification can play a pivotal role in boosting consumption by certifying the superior quality of these products [17]. A study of Spanish consumers by [18] found that the PGI label is particularly influential among occasional consumers, though product origin remains the primary determinant of purchase decisions. Consumers often prefer locally sourced products for qualitative reasons (fresher and tastier meat) and moral considerations (supporting the domestic economy) [19].

In addition to origin, other product attributes such as shelf life, cut type, and nutritional information play a significant role in consumer decision-making [20]. Modern consumers are increasingly attentive to the nutritional composition of food due to a focus on healthy lifestyles [21,22] while also valuing the sensory pleasure of meat consumption [23]. Lamb meat is distinguished by its unique organoleptic qualities flavor, taste, and aroma as well as its nutritional profile, which includes essential proteins and fatty acids [24]. Pasture-fed lamb meat has a more intense flavor than that of lambs raised on concentrate or milk, and the slaughter age further affects tenderness and aroma [25]. Lamb is also a rich source of polyunsaturated fatty acids (PUFAs), including conjugated linoleic acid (CLA), arachidonic acid, Omega-3 fatty acids, and essential minerals like iron and zinc [26,27], all of which contribute to human health [28]. Grazing-based production systems, in particular, yield lamb meat with a PUFA-rich fatty acid profile [29].

In an era where red meat consumption is increasingly discouraged for environmental and health reasons [30], the traditional lamb production system, which is largely pasture-based and reliant on transhumance, should not be overlooked. This system produces high-quality food from limited natural resources, supports sustainable land management, and functions as a multifunctional system [31]. However, understanding the offer composition and planning is also important to depict a holistic view of the lamb market in a particular geographic area, characterized by located production areas and seasonal consumption [32].

To provide actionable insights and marketing strategies for sustaining these production systems and addressing the decline in lamb consumption, this research aims to:

1. Identify the profiles of lamb meat consumers (and buyers) and non-consumers, analyzing preferences and purchasing behaviors as well as reasons for non-consumption.
2. Describe and analyze product attributes in large-scale retail (LSR) settings across three time periods: before, during, and after Easter holidays.
3. Examine the relationship between supply and demand to assess whether the production chain aligns with consumer intentions.

In order to reach the latter research aims the Best-Worst Scaling (BWS) methodology was employed to investigate consumer preferences. While this approach has been applied in other agri-food sectors [33,34,35,36,37], its use in lamb consumer research is, to the best of our knowledge, unprecedented. Similarly, studies comparing simultaneously the supply and demand dynamics in the lamb sector are underrepresented in recent literature.

In regions such as the one analyzed, where lamb consumption is historically rooted, data generated by these surveys provide valuable information to operators in the lamb meat supply chain, enabling them to better understand consumer preferences and effectively fill gaps in supply.

## 2. Materials and Methods

### 2.1. Part 1: Analysis of Lamb Meat Demand

#### 2.1.1. Data Collection (Demand Analysis) 

A face-to-face consumer survey was conducted at two points of sale of LSR in the metropolitan area of Turin (Piedmont—Northwest Italy) from September 2022 to July 2023 using a structured questionnaire. The two interview collection points correspond to two hypermarkets, chosen for their availability of lamb meat and high visitor numbers. Prior to participation, respondents were presented with an introductory document outlining the purpose of the research and the structure of the questionnaire. Participants were recruited using a non-probabilistic convenience sampling approach at selected large-scale retail outlets. The only inclusion criterion was being at least 18 years old. No a priori age stratification was applied; age was collected as a socio-demographic variable and used ex post to describe the sample composition rather than to ensure representativeness of the target population. The online survey was conducted anonymously, and respondents were required to provide their consent before participating. This consent came after they had read a disclosure sheet describing the project and survey aims. The questionnaire was developed in Italian. The research adhered to the principles outlined in the Declaration of Helsinki. It took about 8–10 minutes to complete the questionnaire.

**Figure 1:**
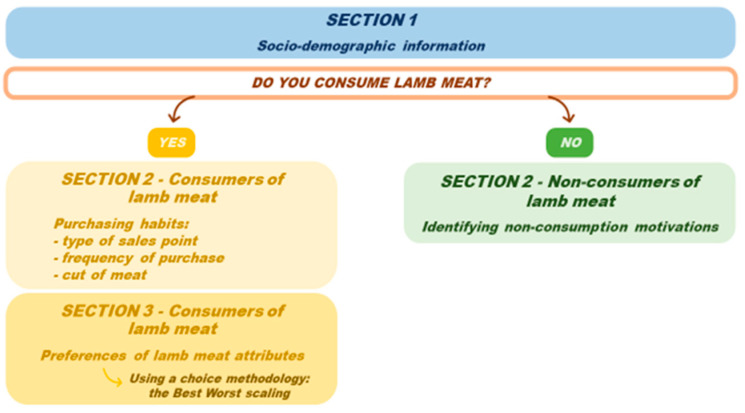
Structure of the questionnaire divided between consumers and non-consumers.

The questionnaire was structured in three sections (Figure 1). Questions (pathways) were differentiated according to respondents’ declaration of consumption or non-consumption of lamb meat.

The first section, common for both types of interviewees, included the socio-demographic variables of individuals (age, gender, family size, education, religion, employment status, annual income). Subsequently, respondents were asked whether they consumed lamb meat, dividing the survey into two questionnaires: one for non-consumers and one for consumers/responsible for meat purchasing. For the non-consumers, the reasons why they do not consume lamb were investigated using a multiple choice (CATA) question (Table 1).

For the lamb meat consumer, the questionnaire continued by defining their purchasing habits (place and time of purchase, preference of cut of meat) [40].

In the last section, the stated preferences of consumer towards a set of lamb meat attributes were collected using the Best-Worst scaling approach. BWS is a methodology used to directly evaluate individual preferences for a set of attributes related to a specific topic [41]. The choice of this methodology over the traditional Likert scale, typically used to gauge agreement or disagreement with specific product attributes, stems from the superior accuracy of responses obtained through BWS. Unlike the Likert scale, which often suffers from respondents’ reluctance to select extreme positive or negative options, BWS effectively mitigates this issue, providing more precise and actionable data [42]. It involves selecting the best and worst options from a proposed list of attributes [43]. In accordance with the experimental design already employed by [35], 12 attributes characterizing lamb meat were identified. These attributes were selected based on an extensive literature review and are detailed in Table 2 [35,43,44].

Beyond their technical construction, the developed scale should be interpreted as analytical tools aimed at capturing underlying consumer perceptions rather than as absolute measures. Higher scores reflect stronger relative importance or agreement with the investigated dimensions, allowing meaningful comparison across attributes and consumer heterogeneity. Therefore, the interpretative value of the scales lies primarily in their ability to reveal preference structures and trade-offs, rather than in the numerical magnitude of the scores themselves.

The balanced incomplete block design (BIBD) was used to equally distribute the various attributes in random combinations within the choice tasks. Each participant had to complete a total of 9 sets (BW questions), each of which contained a subset of 4 (b) attributes randomly selected from the original list of 12 (*n*) attributes [56]. Each item appeared 3 times in the dedicated section, and 4 different versions of the questionnaire were developed in which the order of the attributes within each set changed in order to increase the number of possible pairs of items that respondents could select. For each BW question, was asked to the respondent to select the one alternative they considered the “best” and the one they considered the “worst” in each set (pair of items with maximum preference difference). The repeated choice of the couple of maximum difference of preferences for each BW question allowed for a nuanced understanding of consumer preferences and the relative importance assigned to each meat attribute. The experimental design was generated using Sawtooth experimental design software (SSI version 8.4.6, Orem, UT, USA; http://www.sawtoothsoftware.com/), which maximized statistical efficiency and reduced presentation order bias.

#### 2.1.2. Data Analysis of Consumer Preferences

The whole sample of the respondents was divided in two clusters: consumers (responsible for meat purchasing) and non-consumers [32,57]. The descriptive analysis of socio-demographic characteristics was made comparing the two individuals’ groups.

For lamb meat consumers, the BWS responses were analysed to identify the most significant attributes of lamb meat.

The data gathered from the BWS questions were analysed using Sawtooth Software (SSI-version 8.4.6, Sawtooth Software, Orem, UT, USA) using a discrete choice model. The input matrix for the software was organized as a matrix with n rows, where each row corresponds to a respondent (*n* = sample size), and 18 columns arranged in 9 pairs. Each pair of columns represents one BWS question: the first column in each pair shows the position (from 1 to 4) of the attribute chosen as “BEST” in that particular set (or question), while the second column shows the position of the attribute chosen as “WORST” (also from 1 to 4) [41]. This pattern continues for the remaining 8 pairs, corresponding to the other BWS questions. The matrix also includes an additional column indicating the version (from 1 to 4) of the questionnaire completed by each respondent. The Hierarchical Bayesian (HB) approach was used to analyze the Best-Worst Scaling responses employing a logit model, commonly used in the HB context, in order to estimate preferences. Within the Hierarchical Bayesian framework, preferences are modeled at two levels. At the individual level, each respondent is characterized by a vector of utility parameters reflecting their specific preference structure. These individual-level parameters are assumed to be random draws from a population-level multivariate distribution, which represents overall preferences in the sample. Overall preferences are then derived by aggregating individual posterior utility estimates, and summarized through the Average Raw Score (ARS), which represents the mean importance of each attribute at the population level [58]. The HB algorithm employs an iterative sampling procedure known as Monte Carlo Markov Chain (MCMC) to generate samples from a posteriori distribution of individual preferences. At each iteration, individual preferences and the overall population distribution are estimated, continuously refining the estimates until convergence is reached. After MCMC sampling convergence, we obtain individual utility estimates for each attribute or alternative [59]. These utility scores represent individual preferences and can be aggregated to obtain a sample-level estimate average raw score (ARS). This aggregation makes it possible to identify overall trends in the sample in relation to the expressed preferences. The ARS could be negative and positive. This first result then provides an individual preference index, as well as an average preference index calculated over the entire population that was used to rank the preferences of the 12 selected items.

### 2.2. Part 2: Analysis of Lamb Meat Supply

#### 2.2.1. Data Collection (Supply Analysis) 

For the study of the attributes of lamb meat in the LSR, an exploration of the lamb meat supply was conducted directly in the 56 points of sales of the metropolitan area of Turin (Piedmont—North-West Italy), the same research area as the demand survey. The 56 sales outlets included in the supply analysis were selected using a purposive, non-probabilistic sampling approach. Selection criteria were defined ex ante to ensure coverage of the main large-scale retail formats (hypermarkets, supermarkets, discount stores, and proximity outlets) and the major retail chains operating in the metropolitan area of Turin. The same outlets were monitored across the three survey periods to ensure temporal comparability of supply characteristics. Therefore, the sample is not statistically representative of all LSR outlets, but it allows for an in-depth and consistent analysis of supply dynamics within the study area. In particular from studies of the supply chain [60], 7 attributes with different levels were selected and explored (Table 3).

Data collection was conducted by checking the products displayed in large retail chains during three different periods. The decision to compare and study the temporal evolution of the selected variables is linked to the seasonality of lamb consumption in Italy, which is highest during the Easter holidays. Each period corresponds to 20 days of research, carried out during 2023: (a) pre-Easter (February-March), (b) Easter (April) and (c) post-Easter (May).

#### 2.2.2. Data Analysis of Lamb Meat Supply

The statistical analysis of the attributes relating to the supply of lamb in the LSR was carried out in two elaborations: a Correspondence analysis (CA) and a Univariate analysis of variance (general linear ANOVA-GLM models). Table 4 summaries which variables were used for the first and second statistical models.

##### Correspondence Analysis

The Correspondence analysis (CA) was conducted to explore the association between the levels of the product attributes (Table 4) with the three selected periods of the year.

CA was already used to analyze the relationship by the characteristics of a product and the consumer preferences [32]. However, to the best of our knowledge, it has not yet been used in the case of lamb. This analysis first involves the creation of a contingency table whose rows correspond to the levels of each attribute (categorical variables), while the columns were the three levels (Before-Easter, Easter and After-Easter) of the period variable (nominal variables) [63]. The CA uses the frequencies emerging from the contingency table as graphical points in a geometric space: based on Chi-square distances and whose axes correspond to the identified principal components [64,65]. In the graph, therefore, the points that are closer together will indicate a stronger connection between variables from both rows and columns [66]. CA was carried out using R studio software version 4.3.2.

##### Univariate Analysis of Variance

Some of the selected variables already used in the CA (Table 4) were analysed by comparing the groups of variables according to price using the analysis of variance [67]. This elaboration was conducted using general linear ANOVA models. Price was used as dependent variable, while the variables period, shop format, origin and Protected Geographical Indication (PGI) were used as independent variables. These variables were thus analysed to test the main effect and interaction effects between the independent variables on average prices [63]. The ANOVA was performed using SPSS 27.0 for Windows.

## 3. Results

### 3.1. Part 1: Evidences About Lamb Meat Demand Characterization 

#### 3.1.1. Socio-Demographic Description

The Table 5 shows the socio-demographic characteristics of the total of 347 individuals interviewed for this research. Of the total, 135 respondents (39%) declared themselves to be consumers of lamb. On the contrary, 212 (61%) were non-consumers of lamb.

Table 5 highlights notable differences in socio-demographic characteristics between the total sample, lamb consumers, and non-consumers. The sample is predominantly male (75.4%), with men more likely to be lamb consumers (77.7%) compared to women (22.3%). This imbalance is likely related to the survey setting and to the focus on individuals responsible for meat purchasing. Most respondents identify as Christian (68.8%), with higher proportions among consumers (76.2%) and non-consumers (80.6%). Notably, a significant share of atheists is observed in the total sample (28.1%), though fewer atheists are consumers (21.4%) than non-consumers (15.5%).

Higher educational levels are prevalent among lamb consumers, with 44.2% holding a master’s degree and only 2.4% with post-graduate qualifications. This contrasts with non-consumers, where 39.8% hold a master’s degree, but more (7.5%) have post-graduate degrees. Younger individuals (18–25 years) dominate the consumer group (48.3%), suggesting stronger interest in lamb among students or young adults. Conversely, older age groups (>65 years) are more represented among non-consumers (16.9%).

Lamb consumption appears higher among larger households, as 38.7% of consumers live in households with four members, compared to 30.2% of non-consumers.

Students are the largest group among consumers (46.2%), highlighting a younger demographic, while employees dominate the non-consumer group (31.3%). Retirees also represent a higher share of non-consumers (18.8%). Consumers are concentrated in the lower income brackets (<€25,000, 38.8%), with relatively few earning >€60,000 (2.4%). Non-consumers exhibit a more balanced income distribution, with notable representation in the €25,000–40,000 range (28.3%).

A significant portion of consumers (21.6%) and non-consumers (17.1%) prefer not to disclose their annual income, indicating a potential sensitivity or hesitancy in sharing financial information.

#### 3.1.2. Motivations for Non-Consumption

The perception of an unpleasant taste was the main reason (47.6%) for non-consumption of lamb among the selected individuals’ sample. The low familiarity (28.3%) and the ethic motivations (22.1%) represented other important drivers for the not-consumption of lamb meat.

#### 3.1.3. Lamb Consumption and Purchasing Style

The consumer survey also explored lamb purchasing and consumption patterns, focusing on three key aspects: the main purchasing channels, the preferred periods for lamb consumption, and the most commonly chosen meat formats.

##### Preferred Purchasing Channels

The results revealed that butcher shops are the most popular purchasing channel for lamb, with 42.9% of consumers selecting their trusted local butcher. However, supermarkets and hypermarkets were nearly as popular, accounting for 38.5% of responses. These findings suggest a balance between the convenience offered by large retailers and the perceived quality and trust associated with local butcher shops.

##### Seasonality of Lamb Consumption

Regarding the periods during which lamb is consumed or purchased, the frequency analysis indicates that, contrary to traditional expectations, consumers in the study area do not predominantly associate lamb consumption with holidays and festivities. Instead, 31.8% of respondents reported consuming lamb throughout the year. This shift in consumption patterns may reflect changing consumer habits, including a departure from traditional seasonal norms towards more flexible and year-round preferences for lamb. Further analysis could investigate whether these trends are linked to cultural changes, availability, or evolving dietary habits.

##### Preferred Meat Formats

Consumer preferences for lamb meat formats are diverse, but bracelets (35.5%), lombed (30.3%), and whole (22.2%) emerged as the most popular choices. The remaining meat formats showed relatively similar frequencies, indicating a wide range of preferences among consumers. These results align with existing studies on meat consumption, which suggest that cuts offering versatility and ease of preparation tend to be favored by consumers.

#### 3.1.4. Best-Worst Scaling

To address the first research question regarding consumer preferences, the degree of importance attributed to 12 specific product attributes (Table 2) was analyzed. As shown in Table 6, ‘Origin’ emerged as the most influential attribute, with the highest ARS (1.918). This attribute was selected as “Best” the highest number of times (206) and as “Worst” the fewest times (39). The second most influential attribute was ‘Cut of Meat’, with an ARS of 1.629, followed by ‘Price’, which had an ARS of 0.864.

Conversely, the attributes considered least influential, as indicated by the lowest ARS values, were Ease of Preparation (−0.971), Promotional Offers (−1.227) and Occurrence of Religious Holiday (−1.227).

These attributes were most frequently selected as “Worst” by respondents, indicating their minimal influence on purchasing decisions.

### 3.2. Part 2: Lamb Meat Market

#### 3.2.1. Correspondence Analysis

A total of 449 references were obtained from 56 points of sale of 21 different large retail chains. During each survey period—Before, During and After Easter—data were collected from the same sales locations. This allowed an early analysis to discover that in many shops the product was only present during the festive period.

##### Marketing Attributes: Number of References, Retail Outlet Format, Private Label, Price Discount, and Offer

**Figure 2:**
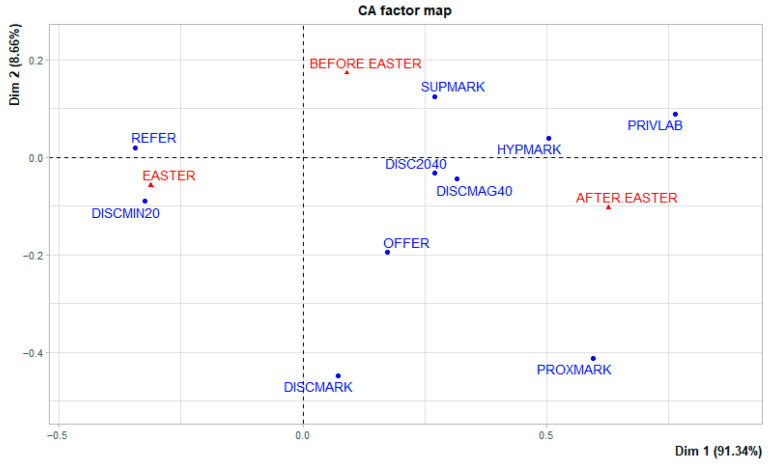
Correspondence analysis of marketing attribute. REFER = references; PRIVLAB = private label; OFFER = promotional offer; DISCMIN20 = discount less than 20%; DISC2040 = discount between 20 and 40%; DISCMAG40 = discount greater than 40%; HYPMARK = hypermarket; SUPMARK = supermarket; PROXMARK = proximity market; DISCMARK = discount market.

The results of the Correspondence analysis of marketing attributes with the three periods (Before Easter, Easter, After Easter) in which the case study was focused are shown in Figure 2.

The graph highlights that in the pre-Easter period lamb is mainly found in hypermarkets and supermarkets while in the post-Easter period the product is located in discount and proximity shops. During the Easter period, the product is present in all types of LSR sales points and the number of references, the number of products detected, is the highest.

In terms of price discounting, a 20% discount was most associated with the festive period. Discounts above 20% are associated with the last survey period. In fact, this is confirmed by the fact that product offers were found in the period after Easter.

Finally, the private label attribute was detected more during the first survey period.

##### Lamb Cuts Variety

**Figure 3:**
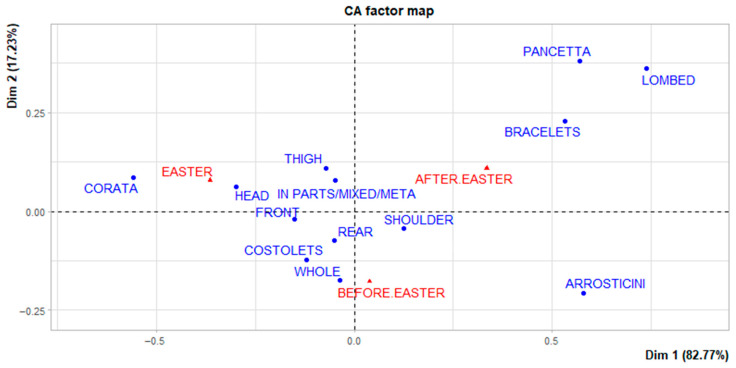
Correspondence analysis of lamb cuts.

In the case of the types of meat cuts, some differences related to the period of detection can also be seen from the graph (Figure 3). In particular, the *arrosticini* format was detected more during the pre-Easter period. The *corata*, on the other hand, is more associated with the Easter period. While after the festive season, the cuts *pancetta*, *lombed* and *bracelets* were more present.

##### Origin and PGI

**Figure 4:**
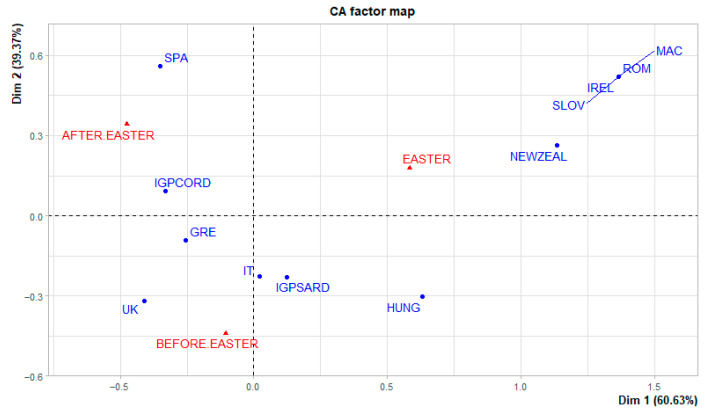
Correspondence analysis of origin and PGI. IT = Italy; SPA = Spain; GRE = Greece; UK = United Kingdom; IREL = Ireland; NEWZEAL = New Zealand; HUNG = Hungary; SLOV = Slovakia; ROM = Romania; MAC = Macedonia; IGPSARD = PGI *Agnello Sardo* (Italy); IGPCORD = PGI *Cordero De Extramadura* (Spain).

The graph in Figure 4 reports the correspondence analysis of product origin attributes and Protected Geographical Indication (PGI) certification. The results indicate that during the Easter period there is the greatest presence of imported products from abroad, in particular from: Eastern Europe (Romania, Macedonia, Slovenia), Ireland and New Zealand. Hungarian lamb, on the other hand, can be associated with both the Easter period and the previous period.

The origin of the Italian, Greek and British product is associated with the first survey period while the Spanish product with the third period.

The PGI attribute confirms what was found for the product origin: the PGI of Sardinian lamb corresponds to Italian origin and the PGI of Cordero De Extremadura is associated with Spanish origin.

#### 3.2.2. Univariate Analysis of Variance

Descriptive statistics, carried out prior to the univariate analysis, provide an overview of average product prices according to the period of research, shop format, origin and the presence of PGI certification (table in Appendix A). In general, the total average price is highest in the period after Easter (13.64 euro), followed by the period before Easter (13.38 euro). The lowest average price emerged in the festive period (11.82 euro).

Table 7 shows some significant differences between the average prices found for lamb: in the different sales outlets, of different origins, and with PGI certification. In particular, the average price of lamb at the discount shop is significantly different from the same product in the other LSR outlets. Similarly, origin showed significant differences in the average prices of products from: Italy, the UK, New Zealand and Ireland. Finally, products without PGI certification showed a significantly different average price than products with certification.

The univariate analysis was composed of additional data, which for the sake by completeness are listed in Appendix B.

## 4. Discussion

This research provides valuable insights into consumer behaviour and supply dynamics within the large-scale retail (LSR) sector, specifically about lamb meat. Specifically, in the metropolitan area of Turin. By analysing consumer preferences and comparing them with the attributes of products available in LSR, the study reveals areas of alignment and mismatch between demand and supply.

### 4.1. Consumer Preferences

The analysis of consumer preferences revealed that origin is the most important attribute influencing lamb meat purchases, followed by the cut of meat and price. This aligns with previous studies suggesting that consumers value locally sourced products for their perceived quality, freshness, and cultural relevance [74,75,76,77]. The preference for high-quality cuts reflects a general trend in meat consumption, where consumers seek versatility and ease of preparation [78]. In addition, the consumer sample was characterized by a higher proportion of male subjects. Previous studies indicate that men tend to exhibit higher meat consumption and lower ethical sensitivity than women [79,80].

Interestingly, attributes such as ease of preparation, promotional offers and seasonality were less influential. This suggests that, although lamb is often associated with festive occasions, regular consumers view it as a year-round product and prioritize intrinsic qualities over external factors such as price discounts or seasonal promotions. These findings challenge the traditional perception of lamb as a niche product associated with specific events [81].

### 4.2. Supply Dynamics in LSR

The supply analysis revealed that the availability of lamb in LSR is strongly influenced by the holiday season, with a significant variety and quantity of products, including an increase in meat from non-European countries, during Easter. This probably results in a lower average product price during the festive season compared to the periods before and after. The focus on festive periods may be driven by the seasonal nature of sheep farming, with production cycles traditionally aligning with demand spikes during holidays [82]. However, the growing trend toward de-seasonalization in livestock production offers opportunities to better meet consumer expectations [83].

The study also found that imported lamb dominates during the holiday season, while products of Italian origin, including those with PGI certification, are more prevalent outside of this period. This could indicate logistical challenges in meeting peak demand with domestic supply, or it could suggest that PGI-certified products are positioned as a niche market [74]. Furthermore, promotional offers and private-label products, primarily used to drive sales post-holiday, appear to have limited influence on consumer purchasing decisions, underscoring the importance of aligning supply strategies with consumer priorities.

### 4.3. Mismatch Between Demand and Supply

There is a mismatch between consumer preferences and LSR supply. LSR focuses on holiday availability and promotions, but consumers want consistent, year-round access to high-quality lamb. Retailers can adjust supply chains and marketing to focus on attributes like origin, sustainability and traceability, which resonate more strongly with consumers [54,84].

Additionally, the underrepresentation of organic certifications in LSR, despite their moderate importance to consumers, suggests a potential area for product diversification. Enhancing the visibility and availability of certified organic lamb could appeal to environmentally conscious consumers and address growing concerns about sustainability in meat production [85,86]. However, there are several economic barriers limiting the development of certified organic lamb production: (a) low initial profitability [87] and (b) high consumer cost [86].

## 5. Limitations and Future Research

This study has some limitations:

The sample size is small and is not representative of either the Italian population or the population of Piedmont. It should be noted that lamb consumers account for approximately 39% of the total number of individuals involved. However, this is a non-probabilistic sample of customers from two hypermarkets who were willing to participate in the survey. In particular, a further limitation of this study is the gender imbalance of the sample, with males being overrepresented compared to the regional population; this may have influenced the observed preference structure, particularly with respect to ethical and animal welfare-related attributes, and therefore results should be interpreted with caution and not generalized to the entire population.

Furthermore, the study area was concentrated in the Turin metropolitan area, making it possible to compare a sample of consumers in the same area where the stores involved in the second part of the survey are located. This limits the study, confining it to a single area of Italy.

In the future, it will be necessary to expand the sample of respondents, through interviews conducted not only face to face but also with the support of provider companies, and the study area in order to determine whether the misalignment of supply and demand is widespread throughout Italy or limited to certain areas. Furthermore, following the results obtained, it would be useful to involve all stakeholders in the supply chain through a participatory approach to find practical strategic solutions to align the supply and demand of lamb meat.

## 6. Conclusions

This research underscores the importance of aligning lamb meat supply strategies with evolving consumer preferences. While LSR effectively addresses certain consumer demands, such as variety and origin labeling, there is significant room for improvement in providing year-round availability and catering to preferences for sustainably produced and certified products. Bridging these gaps could enhance consumer satisfaction, drive sales, and support the sustainability of the lamb production chain.

Future studies should explore regional variations in consumer behavior, the role of digital marketing in promoting lamb meat attributes, and the economic feasibility of adapting supply chains to meet year-round demand. These insights will be essential for stakeholders aiming to balance consumer needs with sustainable production practices.

## Figures and Tables

**Table 1:** Motivations for non-consumption.

Motivations for Non-Consumption	References
Disagreeable taste	[4];
Overly fatty	[4];
No environmentally sustainable	[29,38,39];
Ethical reasons it is not right to feed and slaughter a sentient being/animal welfare	[29,39];
No practical to prepare/cook	[4,12];
No consumed at home by anyone else	[12];
Overly expensive	[4,12,38];

**Table 2:** Attribute of the Best-Worst scaling for the research on lamb meat.

Attribute BWS	References
Price	[18,19,45];
Offers	[33,36];
PGI Protected Geographical Indication	[5,18,45,46];
Origin indication of the place where the animals were farmed/slaughtered/sectioned	[19,45,47,48,49,50];
Easy to prepare	[5];
Organic certification	[5,18,51];
Occurrence of religious holiday	[6,52,53];
Age of animal	[5];
Label	[20];
Cut of meat	[35,54];
Fat content (visible)	[3,42];
Availability/Reportability at sales locations	[55]

**Table 3:** Attributes and attribute levels explored in LSR.

Attribute	Levels	References
Price (/kg)	Continuous variable	[18,19,45];
Discount percentage	Not applied Minor than 20% (equal to 20%) Between 20–40% (equal to 40%) Major than 40%	[61];
Private label	Absence Presence	[62];
Cut of meat	Front Rear Head *Corata* Lombed Arrosticini Costolets Bracelets Pancetta In parts/mixed/meta Whole Shoulder Thigh	[54];
PGI Protected Geographical Indication	*Agnello Sardo IGP* *Cordero de Extremadura IGP* Not indicated	[5,18,45,46];
Origin indication of the place where the animals were farmed/slaughtered/sectioned	Italy United Kingdom Spain Eastern European countries (Greece, Romania, Macedonia, Hungary, Slovakia) New Zealand Ireland Not indicated	[19,45,47,48,49,50];
LSR format Large-scale retail sales point format	Hypermarket Supermarket Superette/Convenience store/Proximity service Discount	[32];

**Table 4:** Variables and statistical methods used for the supply analysis.

Variables	Statistical Analysis
Period	CA and GLM
Price (/kg)	GLM
Discount percentage	CA
Private label	CA
Cut of meat	CA
PGI	CA and GLM
Origin	CA and GLM
LSR format	CA and GLM

**Table 5:** Socio-demographic characteristics of the total sample, consumer sample and non-consumer sample.

Socio-Demographic Variables	Item	% Total Sample Interviewed	% Consumer Sample	% Non-Consumer Sample	% Piedmontese Sample ^1^	References
Gender	Women	24.60	22.30	26.90	51.1	[68]
	Men	75.40	77.70	73.10	48.9	[68]
Religion	Christian	68.80	76.20	80.60	n.d.	
	Orthodox	2.30	2.20	2.30	n.d.	
	Protestant	0.20	_	0.40	n.d.	
	Jewish	0.20	_	0.40	n.d.	
	Islamic	0.20	_	0.40	n.d.	
	Jehovah’s Witness	0.20	_	0.40	n.d.	
	Atheist	28.10	21.40	15.50	n.d.	
Education level	Primary school	7.10	7	3.70	33.4	
	Lower secondary school	15.80	12	14.60	n.d.	
	Upper Secondary school	34.80	34.40	34.40		
	Master’s degree	33.50	44.20	39.80	n.d.	
	Post-graduate degree	8.80	2.40	7.50		
Age	18–25	31.60	48.30	26.60	Range 15–64 years 61.9	[69]
	26–35	13	11.80	12.70		
	36–45	10.50	8.10	9.90		
	46–55	15.50	13.30	17.40		
	56–65	14.70	11.10	16.50		
	>65	14.70	7.40	16.90	26.9	[69]
Number of household members	1	13.20	8.10	11.80	39.5	[70]
	2	25.60	16.20	28.30	29.5	[70]
	3	21.90	23.70	25	~31	[70]
	4	31.60	38.70	30.20		
	>4	7.70	13.30	4.70		
Employment status	Student	22.10	46.20	19.30	45	[71,72]
	Employee	26.10	11.80	31.30	48.6 (Employment rate in Piedmont)	[73]
	Self-employed	8.70	5.10	9.90	n.d	
	Retired	17.40	11.10	18.80	45	[72]
	Job seeker	3.40	3.70	4.70	6.1	[73]
	Homemaker	12.10	9.60	9.40	45	[72]
	Other	10.20	12.50	6.60		
Annual income range	<25,000 euro	30.30	38.80	31.60	n.d.	
	25,000–40,000 euro	23.20	22.80	28.30	n.d.	
	40,000–60,000 euro	28.20	14.40	18.80	n.d.	
	>60,000 euro	12.10	2.40	4.20	n.d.	
	I prefer do not respond	6.20	21.60	17.10		

^1^ Regional reference data for education level are reported only where classification is directly comparable with the questionnaire categories.

**Table 6:** Consumer declared preferences towards lamb meat.

Attributes	Times Selected BEST	Times Selected WORST	St. Deviation	Average Raw Score
Origin	206.0	39.0	1.742	1.918
Cut of meat	200.0	44.0	1.339	1.629
Price	133.0	67.0	1.697	0.864
Organic certification	123.0	88.0	1.952	0.504
Age of animal	113.0	70.0	1.274	0.396
Reportability at sales locations	64.0	86.0	0.844	−0.192
Fat content	95.0	123.0	1.756	−0.207
PGI—Protected Geographical Indication	68.0	108.0	1.622	−0.621
Label	41.0	129.0	1.153	−0.864
Easy to prepare	44.0	135.0	1.320	−0.971
Promotional offers	66.0	163.0	1.862	−1.227
Occurrence of religious holiday	62.0	163.0	2.065	−1.227

**Table 7:** Differences in price averages for each level of the variables considered.

		Average Prices	F	*p*-Value
Period	Before Easter	14.771 ^a^	1.703	0.183
	Easter	12.128 ^a^		
	After Easter	13.234 ^a^		
LSR	Hypermarket	12.994 ^a^	2.514	*
	Supermarket	14.089 ^a^		
	Superette	15.207 ^a^		
	Discount	10.902 ^b^		
Origin	Not indicated	12.105 ^a^	2.508	*
	Italy	13.445 ^a,b^		
	United Kingdom	14.568 ^b^		
	Spain	12.119 ^a^		
	Eastern European countries	12.865 ^a^		
	New Zealand	17.015 ^b^		
	Ireland	9.990 ^c^		
PGI	Not indicated	13.355 ^b^	3.477	*
	*Agnello Sardo* IGP	12.544 ^a^		
	*Cordero de Extremadura* IGP	12.905 ^a^		

Significance level: * *p*-value < 0.05. ^a,b,c^ indicate significant differences among mean prices (α = 0.05, Tukey port-hoc test, pairwise comparison).

## Data Availability

The original contributions presented in this study are included in the article. Further inquiries can be directed to the corresponding author.

## Appendix A

**Table A1:** Average prices of products differentiated by: Period, LSR format, Origin, PGI.

Period	LSR Format	Origin	PGI	Average Price	Standard Deviation	Number
Before-Easter	Hypermarket	Not indicated	Not indicated	14.0830	5.37447	10
			Total	14.0830	5.37447	10
		Italy	Not indicated	14.1820	4.97132	5
			Agnello Sardo IGP	14.1257	5.28507	14
			Total	14.1405	5.06614	19
		United Kingdom	Not indicated	13.4156	3.76263	9
			Total	13.4156	3.76263	9
		Spain	Not indicated	14.4450	3.59917	2
			Cordero de Extremadura IGP	13.6750	4.43549	8
			Total	13.8290	4.10444	10
		Eastern European countries	Not indicated	14.3620	3.20353	5
			Total	14.3620	3.20353	5
		Total	Not indicated	13.9735	4.20485	31
			Agnello Sardo IGP	14.1257	5.28507	14
			Cordero de Extremadura IGP	13.6750	4.43549	8
			Total	13.9687	4.45553	53
	Supermarket	Not indicated	Not indicated	10.5885	3.66744	13
			Total	10.5885	3.66744	13
		Italy	Not indicated	12.1069	4.72897	32
			Agnello Sardo IGP	11.9690	3.52465	10
			Total	12.0740	4.43163	42
		United Kingdom	Not indicated	17.4583	2.84114	6
			Total	17.4583	2.84114	6
		Eastern European countries	Not indicated	21.4650	2.14253	2
			Total	21.4650	2.14253	2
		New Zealand	Not indicated	22.3400	10.81873	2
			Total	22.3400	10.81873	2
		Total	Not indicated	13.0442	5.36573	55
			Agnello Sardo IGP	11.9690	3.52465	10
			Total	12.8788	5.11784	65
	Superette	Not indicated	Not indicated	16.4000	0.70711	2
			Total	16.4000	0.70711	2
		Total	Not indicated	16.4000	0.70711	2
			Total	16.4000	0.70711	2
	Discount	Not indicated	Not indicated	10.9450	2.76479	2
			Total	10.9450	2.76479	2
		Total	Not indicated	10.9450	2.76479	2
			Total	10.9450	2.76479	2
	Total	Not indicated	Not indicated	12.3396	4.54309	27
			Total	12.3396	4.54309	27
		Italy	Not indicated	12.3873	4.74557	37
			Agnello Sardo IGP	13.2271	4.67211	24
			Total	12.7177	4.69585	61
		United Kingdom	Not indicated	15.0327	3.89558	15
			Total	15.0327	3.89558	15
		Spain	Not indicated	14.4450	3.59917	2
			Cordero de Extremadura IGP	13.6750	4.43549	8
			Total	13.8290	4.10444	10
		Eastern European countries	Not indicated	16.3914	4.42937	7
			Total	16.3914	4.42937	7
		New Zealand	Not indicated	22.3400	10.81873	2
			Total	22.3400	10.81873	2
		Total	Not indicated	13.3922	4.90413	90
			Agnello Sardo IGP	13.2271	4.67211	24
			Cordero de Extremadura IGP	13.6750	4.43549	8
			Total	13.3783	4.79457	122
Easter	Hypermarket	Not indicated	Not indicated	10.5406	4.70683	33
			Total	10.5406	4.70683	33
		Italy	Not indicated	15.3350	4.58314	12
			Agnello Sardo IGP	12.1550	5.53509	22
			Total	13.2774	5.37378	34
		United Kingdom	Not indicated	16.3300	3.10000	4
			Total	16.3300	3.10000	4
		Spain	Not indicated	9.2950	1.24583	4
			Cordero de Extremadura IGP	9.9400	3.76864	10
			Total	9.7557	3.20659	14
		Eastern European countries	Not indicated	10.5520	3.64543	15
			Total	10.5520	3.64543	15
		New Zealand	Not indicated	8.9500	.	1
			Total	8.9500	.	1
		Total	Not indicated	11.6172	4.69384	69
			Agnello Sardo IGP	12.1550	5.53509	22
			Cordero de Extremadura IGP	9.9400	3.76864	10
			Total	11.5683	4.79963	101
	Supermarket	Not indicated	Not indicated	9.5036	4.60303	28
			Total	9.5036	4.60303	28
		Italy	Not indicated	12.5206	4.19830	33
			Agnello Sardo IGP	13.2678	4.27150	18
			Total	12.7843	4.19691	51
		Spain	Not indicated	11.0330	3.50986	30
			Total	11.0330	3.50986	30
		Eastern European countries	Not indicated	11.2532	4.06654	31
			Total	11.2532	4.06654	31
		New Zealand	Not indicated	19.0100	2.90318	10
			Total	19.0100	2.90318	10
		Total	Not indicated	11.7365	4.60363	132
			Agnello Sardo IGP	13.2678	4.27150	18
			Total	11.9203	4.57866	150
	Superette	Not indicated	Not indicated	15.1289	3.85147	9
			Total	15.1289	3.85147	9
		United Kingdom	Not indicated	13.4000	4.94975	2
			Total	13.4000	4.94975	2
		Total	Not indicated	14.8145	3.84788	11
			Total	14.8145	3.84788	11
	Discount	Not indicated	Not indicated	9.4675	2.39973	4
			Total	9.4675	2.39973	4
		Italy	Not indicated	8.6300	0.71393	3
			Total	8.6300	0.71393	3
		Eastern European countries	Not indicated	10.6241	3.20526	17
			Total	10.6241	3.20526	17
		New Zealand	Not indicated	17.7600	0.00000	3
			Total	17.7600	0.00000	3
		Ireland	Not indicated	9.9900	.	1
			Total	9.9900	.	1
		Total	Not indicated	10.9871	3.59748	28
			Total	10.9871	3.59748	28
	Total	Not indicated	Not indicated	10.6482	4.74022	74
			Total	10.6482	4.74022	74
		Italy	Not indicated	12.9810	4.43963	48
			Agnello Sardo IGP	12.6558	4.97640	40
			Total	12.8332	4.66648	88
		United Kingdom	Not indicated	15.3533	3.59935	6
			Total	15.3533	3.59935	6
		Spain	Not indicated	10.8285	3.36007	34
			Cordero de Extremadura IGP	9.9400	3.76864	10
			Total	10.6266	3.43205	44
		Eastern European countries	Not indicated	10.9165	3.71025	63
			Total	10.9165	3.71025	63
		New Zealand	Not indicated	18.0236	3.59620	14
			Total	18.0236	3.59620	14
		Ireland	Not indicated	9.9900	.	1
			Total	9.9900	.	1
		Total	Not indicated	11.7559	4.52462	240
			Agnello Sardo IGP	12.6558	4.97640	40
			Cordero de Extremadura IGP	9.9400	3.76864	10
			Total	11.8174	4.57568	290
After-Easter	Hypermarket	Not indicated	Not indicated	13.6063	3.58374	8
			Total	13.6063	3.58374	8
		Italy	Not indicated	17.8700	1.24451	2
			Total	17.8700	1.24451	2
		United Kingdom	Not indicated	9.4900	.	1
			Total	9.4900	.	1
		Spain	Not indicated	12.4225	2.11787	4
			Cordero de Extremadura IGP	15.1000	4.70319	3
			Total	13.5700	3.41530	7
		Total	Not indicated	13.5847	3.42061	15
			Cordero de Extremadura IGP	15.1000	4.70319	3
			Total	13.8372	3.54624	18
	Supermarket	Not indicated	Not indicated	6.9900	.	1
			Total	6.9900	.	1
		Italy	Not indicated	19.3350	4.37393	4
			Agnello Sardo IGP	9.8450	5.57907	2
			Total	16.1717	6.45910	6
		United Kingdom	Not indicated	19.9800	.	1
			Total	19.9800	.	1
		Spain	Not indicated	11.0400	3.62974	6
			Total	11.0400	3.62974	6
		Eastern European countries	Not indicated	13.9000	.	1
			Total	13.9000	.	1
		Total	Not indicated	14.2125	5.83131	12
			Agnello Sardo IGP	11.1967	4.58738	3
			Total	13.6093	5.59312	15
	Superette	Not indicated	Not indicated	15.9000	1.41421	2
			Total	15.9000	1.41421	2
		Total	Not indicated	15.9000	1.41421	2
			Total	15.9000	1.41421	2
	Discount	United Kingdom	Not indicated	11.9000	.	1
			Total	11.9000	.	1
		Eastern European countries	Not indicated	7.9000	.	1
			Total	7.9000	.	1
		Total	Not indicated	9.9000	2.82843	2
			Total	9.9000	2.82843	2
	Total	Not indicated	Not indicated	13.4218	3.81871	11
			Total	13.4218	3.81871	11
		Italy	Not indicated	18.8467	3.51580	6
			Agnello Sardo IGP	9.8450	5.57907	2
			Total	16.5962	5.53528	8
		United Kingdom	Not indicated	13.7900	5.49446	3
			Total	13.7900	5.49446	3
		Spain	Not indicated	11.5930	3.05356	10
			Cordero de Extremadura IGP	15.1000	4.70319	3
			Total	12.4023	3.61179	13
		Eastern European countries	Not indicated	13.9000	.	2
			Total	13.9000	.	2
		Total	Not indicated	13.7394	4.43365	31
			Agnello Sardo IGP	11.1967	4.58738	3
			Cordero de Extremadura IGP	15.1000	4.70319	3
			Total	13.6435	4.41165	37
Total	Hypermarket	Not indicated	Not indicated	11.7161	4.87649	51
			Total	11.7161	4.87649	51
		Italy	Not indicated	15.2984	4.41548	19
			Agnello Sardo IGP	12.9214	5.45036	36
			Total	13.7425	5.20137	55
		United Kingdom	Not indicated	13.9679	3.79478	14
			Total	13.9679	3.79478	14
		Spain	Not indicated	11.5760	2.81280	10
			Cordero de Extremadura IGP	12.1000	4.49043	21
			Total	11.9310	3.98475	31
		Eastern European countries	Not indicated	11.5045	3.84936	20
			Total	11.5045	3.84936	20
		New Zealand	Not indicated	8.9500	.	1
			Total	8.9500	.	1
		Total	Not indicated	12.5090	4.52201	115
			Agnello Sardo IGP	12.9214	5.45036	36
			Cordero de Extremadura IGP	12.1000	4.49043	21
			Total	12.5454	4.70383	172
	Supermarket	Not indicated	Not indicated	9.7795	4.28239	42
			Total	9.7795	4.28239	42
		Italy	Not indicated	12.7238	4.70145	69
			Agnello Sardo IGP	12.6067	4.06964	30
			Total	12.6883	4.49902	99
		United Kingdom	Not indicated	17.8186	2.76317	7
			Total	17.8186	2.76317	7
		Spain	Not indicated	11.0342	3.47698	36
			Total	11.0342	3.47698	36
		Eastern European countries	Not indicated	11.8721	4.66574	34
			Total	11.9318	4.60765	34
		New Zealand	Not indicated	19.5650	4.38367	12
			Total	19.5650	4.38367	12
		Total	Not indicated	12.2472	4.93432	199
			Agnello Sardo IGP	12.6484	4.00798	31
			Total	12.3013	4.81403	230
	Superette	Not indicated	Not indicated	15.4431	3.21854	13
			Total	15.4431	3.21854	13
		United Kingdom	Not indicated	13.4000	4.94975	2
			Total	13.4000	4.94975	2
		Total	Not indicated	15.1707	3.33855	15
			Total	15.1707	3.33855	15
	Discount	Not indicated	Not indicated	9.9600	2.35927	6
			Total	9.9600	2.35927	6
		Italy	Not indicated	8.6300	0.71393	3
			Total	8.6300	0.71393	3
		United Kingdom	Not indicated	11.9000	.	1
			Total	11.9000	.	1
		Eastern European countries	Not indicated	10.4728	3.17516	18
			Total	10.4728	3.17516	18
		New Zealand	Not indicated	17.7600	0.00000	3
			Total	17.7600	0.00000	3
		Ireland	Not indicated	9.9900	.	1
			Total	9.9900	.	1
		Total	Not indicated	10.9166	3.44206	32
			Total	10.9166	3.44206	32
	Total	Not indicated	Not indicated	11.3284	4.68157	112
			Total	11.3284	4.68157	112
		Italy	Not indicated	13.1264	4.73343	91
			Agnello Sardo IGP	12.7783	4.83838	66
			Total	12.9801	4.76550	157
		United Kingdom	Not indicated	14.9575	3.86020	24
			Total	14.9575	3.86020	24
		Spain	Not indicated	11.1520	3.32209	46
			Cordero de Extremadura IGP	12.1000	4.49043	21
			Total	11.4491	3.71906	67
		Eastern European countries	Not indicated	11.4138	4.09280	72
			Total	11.4483	4.07442	72
		New Zealand	Not indicated	18.5631	4.60271	16
			Total	18.5631	4.60271	16
		Ireland	Not indicated	9.9900	.	1
			Total	9.9900	.	1
		Total	Not indicated	12.3342	4.67418	361
			Agnello Sardo IGP	12.7951	4.80354	67
			Cordero de Extremadura IGP	12.1000	4.49043	21
			Total	12.3920	4.67834	449

## Appendix B

**Table A2:** Main effects and interaction effects between subjects.

Origin	Sum of Type III Squares	df	Quadratic Mean	F	Sig.
Correct model	2704.198 a	49	55.188	3.101	<0.001
Interceptor	9251.961	1	9251.961	519.851	<0.001
Main effects					
PERIOD	60.622	2	30.311	1.703	0.183
LSR	134.223	3	44.741	2.514	0.058
ORIGIN	267.832	6	44.639	2.508	0.021
PGI	123.758	2	61.879	3.477	0.032
Interaction					
PERIOD * LSR	15.732	5	3.146	0.177	0.971
PERIOD * ORIGIN	398.731	8	49.841	2.801	0.005
PERIOD * PGI	136.707	4	34.177	1.920	0.106
LSR * ORIGIN	494.884	9	54.987	3.090	0.001
LSR * PGI	23.956	1	23.956	1.346	0.247
ORIGIN * PGI	0.000	0	.	.	.
PERIOD * LSR * ORIGIN	148.524	5	29.705	1.669	0.141
PERIOD * LSR * PGI	26.032	1	26.032	1.463	0.227
PERIOD * ORIGIN * PGI	0.000	0	.	.	.
LSR * ORIGIN * PGI	0.000	0	.	.	.
PERIOD * LSR * ORIGIN * PGI	0.000	0	.	.	.
Error	7101.131	399	17.797		
Total	78,754.317	449			
Correct total	9805.328	448			

a. R-square = 0.276 (adjusted R-square = 0.187). *: Indicate the interaction between variables.

**Table A3:** Comparison Pairwise Period.

(I) Period	(J) Period	Difference of the Mean (I–J)	Std. Error	Sig. d	95% Confidence Interval for Difference d	
					Lower Bound	Upper Bound
Before Easter	Easter	2.643 *.b.c	0.693	<0.001	1.279	4.006
	After Easter	1.537 b.c	1.037	0.139	−0.501	3.574
Easter	Before Easter	−2.643 *.b.c	0.693	<0.001	−4.006	−1.279
	After Easter	−1.106 b.c	0.986	0.263	−3.045	0.833
After Easter	Before Easter	−1.537 b.c	1.037	0.139	−3.574	0.501
	Easter	1.106 b.c	0.986	0.263	−0.833	3.045

Based on estimated marginal averages: *. The difference in the mean is significant at the 0.05 level. b. An estimate of the modified population marginal mean (I). c. An estimate of the marginal mean of the modified population (J). d. Adjustment for multiple comparisons: least significant difference (equivalent to no adjustment).

**Table A4:** Comparison Pairwise LSR.

(I) LSR	(J) LSR	Difference of the Mean (I–J)	Std. Error	Sig. d	95% Confidence Interval for Difference d	
					Lower Bound	Upper Bound
Hypermarket	Supermarket	−1.095 a.b	0.738	0.138	−2.546	0.355
	Superette	−2.213 a.b	1.425	0.121	−5.014	0.587
	Discount	2.092 a.b	1.217	0.087	−0.302	4.485
Supermarket	Hypermarket	1.095 a.b	0.738	0.138	−0.355	2.546
	Superette	−1.118 a.b	1.449	0.441	−3.966	1.730
	Discount	3.187 a.b.*	1.245	0.011	0.739	5.635
Superette	Hypermarket	2.213 a.b	1.425	0.121	−0.587	5.014
	Supermarket	1.118 a.b	1.449	0.441	−1.730	3.966
	Discount	4.305 a.b.*	1.743	0.014	0.879	7.731
Discount	Hypermarket	−2.092 a.b	1.217	0.087	−4.485	0.302
	Supermarket	−3.187 a.b.*	1.245	0.011	−5.635	−0.739
	Superette	−4.305 a.b.*	1.743	0.014	−7.731	−0.879

Based on estimated marginal averages: *. The difference in the mean is significant at the 0.05 level. a. An estimate of the modified population marginal mean (I). b. An estimate of the marginal mean of the modified population (J). d. Adjustment for multiple comparisons: least significant difference (equivalent to no adjustment).

**Table A5:** Comparison Pairwise Origin.

(I) Origin	(J) Origin	Difference of the Mean (I–J)	Std. Error	Sig. d	95% Confidence Interval for Difference d	
					Lower Bound	Upper Bound
Not indicated	Italy	−1.340 a.b	0.864	0.122	−3.039	0.359
	United Kingdom	−2.463 a.b	1.392	0.078	−5.200	0.274
	Spain	−0.014 a.b	0.982	0.989	−1.944	1.916
	Eastern European countries	−0.760 a.b	1.230	0.537	−3.178	1.658
	New Zealand	−4.910 a.b.*	1.620	0.003	−8.096	−1.725
	Ireland	2.115 a.b	4.275	0.621	−6.289	10.518
Italy	Not indicated	1.340 a.b	0.864	0.122	−0.359	3.039
	United Kingdom	−1.123 a.b	1.317	0.395	−3.712	1.467
	Spain	1.326 a.b	0.872	0.129	−0.389	3.041
	Eastern European countries	0.580 a.b	1.145	0.613	−1.670	2.830
	New Zealand	−3.570 a.b.*	1.556	0.022	−6.630	−0.510
	Ireland	3.455 a.b	4.251	0.417	−4.902	11.812
United Kingdom	Not indicated	2.463 a.b	1.392	0.078	−0.274	5.200
	Italy	1.123 a.b	1.317	0.395	−1.467	3.712
	Spain	2.449 a.b	1.397	0.080	−0.298	5.195
	Eastern European countries	1.703 a.b	1.581	0.282	−1.406	4.811
	New Zealand	−2.447 a.b	1.901	0.199	−6.184	1.290
	Ireland	4.578 a.b	4.389	0.298	−4.050	13.205
Spain	Not indicated	0.014 a.b	0.982	0.989	−1.916	1.944
	Italy	−1.326 a.b	0.872	0.129	−3.041	0.389
	United Kingdom	−2.449 a.b	1.397	0.080	−5.195	0.298
	Eastern European countries	−0.746 a.b	1.236	0.546	−3.176	1.683
	New Zealand	−4.896 a.b.*	1.625	0.003	−8.090	−1.702
	Ireland	2.129 a.b	4.276	0.619	−6.278	10.536
Eastern European countries	Not indicated	0.760 a.b	1.230	0.537	−1.658	3.178
	Italy	−0.580 a.b	1.145	0.613	−2.830	1.670
	United Kingdom	−1.703 a.b	1.581	0.282	−4.811	1.406
	Spain	0.746 a.b	1.236	0.546	−1.683	3.176
	New Zealand	−4.150 a.b.*	1.786	0.021	−7.660	−0.639
	Ireland	2.875 a.b	4.340	0.508	−5.657	11.407
New Zealand	Not indicated	4.910 a.b.*	1.620	0.003	1.725	8.096
	Italy	3.570 a.b.*	1.556	0.022	0.510	6.630
	United Kingdom	2.447 a.b	1.901	0.199	−1.290	6.184
	Spain	4.896 a.b.*	1.625	0.003	1.702	8.090
	Eastern European countries	4.150 a.b.*	1.786	0.021	0.639	7.660
	Ireland	7.025 a.b	4.466	0.117	−1.755	15.805
Ireland	Not indicated	−2.115 a.b	4.275	0.621	−10.518	6.289
	Italy	−3.455 a.b	4.251	0.417	−11.812	4.902
	United Kingdom	−4.578 a.b	4.389	0.298	−13.205	4.050
	Spain	−2.129 a.b	4.276	0.619	−10.536	6.278
	Eastern European countries	−2.875 a.b	4.340	0.508	−11.407	5.657
	New Zealand	−7.025 a.b	4.466	0.117	−15.805	1.755

Based on estimated marginal averages: *. The difference in the mean is significant at the 0.05 level. a. An estimate of the modified population marginal mean (I). b. An estimate of the marginal mean of the modified population (J). d. Adjustment for multiple comparisons: least significant difference (equivalent to no adjustment).

**Table A6:** Comparison Pairwise PGI.

(I) PGI	(J) PGI	Difference of the Mean (I–J)	Std. Error	Sig. d	95% Confidence Interval for Difference d	
					Lower Bound	Upper Bound
Not indicated	Agnello Sardo IGP	0.811 a.b	1.016	0.425	−1.186	2.808
	Cordero de Extremadura IGP	0.450 a.b	1.122	0.689	−1.756	2.656
Agnello Sardo IGP	Not indicated	−0.811 a.b	1.016	0.425	−2.808	1.186
	Cordero de Extremadura IGP	−0.361 a.b	1.407	0.798	−3.128	2.405
Cordero de Extremadura IGP	Not indicated	−0.450 a.b	1.122	0.689	−2.656	1.756
	Agnello Sardo IGP	0.361 a.b	1.407	0.798	−2.405	3.128

Based on estimated marginal averages: a. An estimate of the marginal mean of the modified population (I). b. An estimate of the modified population marginal mean (J). d. Adjustment for multiple comparisons: least significant difference (equivalent to no adjustment).
